# Building bridges between fields: bringing together development and homeostasis

**DOI:** 10.1242/dev.193268

**Published:** 2021-07-19

**Authors:** Sonja D. C. Weterings, Marek J. van Oostrom, Katharina F. Sonnen

**Affiliations:** Hubrecht Institute-KNAW (Royal Netherlands Academy of Arts and Sciences) and University Medical Center Utrecht, Utrecht, The Netherlands

**Keywords:** Embryonic development, Patterning, Self-organization, Signalling, Stem cell control, Tissue homeostasis

## Abstract

Despite striking parallels between the fields of developmental biology and adult tissue homeostasis, these are disconnected in contemporary research. Although development describes tissue generation and homeostasis describes tissue maintenance, it is the balance between stem cell proliferation and differentiation that coordinates both processes. Upstream signalling regulates this balance to achieve the required outcome at the population level. Both development and homeostasis require tight regulation of stem cells at the single-cell level and establishment of patterns at the tissue-wide level. Here, we emphasize that the general principles of embryonic development and tissue homeostasis are similar, and argue that interactions between these disciplines will be beneficial for both research fields.

## Abstract (precis)

**Summary:** This Review emphasizes that the general principles of embryonic development and tissue homeostasis are similar, and that interactions between these disciplines will be beneficial for both research fields.

## Introduction

Tight regulation of cellular processes in space and time is essential in multicellular organisms. This is true not only during development, when a whole organism forms from a single cell, but also during homeostasis, when an organism has to be maintained. Stem cell divisions drive both development and homeostasis of tissues (Box 1). When transition from development to homeostasis occurs, stem/progenitor cells similarly progress from developmental progenitors to adult tissue stem cells. Within this transition, the focus shifts from growth to maintenance of tissue. Classically, development and homeostasis have been studied using *in vivo* models such as embryos and adult tissues, respectively. Recent breakthroughs in *in vitro* culture systems and technologies for functional investigation have provided the opportunity to understand development and homeostasis (reviewed by He et al., 2020; Shahbazi et al., 2019). By applying these new approaches to diverse model systems, many similarities between developmental and homeostatic processes have been unveiled. In this Review, we explore these similarities, highlighting general principles that control both development and homeostasis. Parallels can be found in stem cell control and the formation and maintenance of tissue patterns by gradients and signalling dynamics (Box 1). By showcasing the similarities between development and homeostasis, we argue that collaborative interactions between the fields are beneficial for both disciplines and can be translated to other fields, such as regeneration and cancer.

**Box 1. General principles and questions for future research**

**Stem cell control and dynamics**

Regulating the balance between proliferation and differentiation of stem cells determines tissue organization. Stem cell divisions can either be symmetric or asymmetric. When a stem cell divides into two daughter cells, they either maintain their stem cell state, differentiate or one persists as a stem cell while the other differentiates. Division symmetry can be regulated intrinsically by asymmetric distribution of cell fate determinants and/or on a population level by external signalling created by the microenvironment.

Outstanding questions include ‘where and what are tissue-specific progenitors and stem cells?’, ‘what cells make up the stem cell niche?’ and ‘which signals control stem cell behaviour?’.

**Tissue self-organization**

The initial step of self-organization is a symmetry-breaking event. After breaking symmetry, signalling centres are established that guide self-organization processes such as patterning and collective cell rearrangements. Collective cell rearrangements are used to subsequently arrange stem cells and differentiated cells within tissues.

Outstanding questions include ‘what is the molecular mechanism of symmetry breaking?’, ‘is the mechanism similar in different species?’ and ‘what are the signals and intracellular processes guiding collective cell migration?’.

**Pattern formation and maintenance**

Gradients pattern tissues by subdividing tissues into domains of differentiation. Gradients are formed during development and maintained during homeostasis. An additional layer of specificity in tissue patterning is provided by signalling dynamics that orchestrate cell proliferation and differentiation.

Outstanding questions include ‘how are signalling gradients established and maintained?’, ‘how are gradients decoded to control cell fate?’, ‘what is the function of signalling dynamics?’ and ‘how do signalling dynamics control cellular behaviour?’.

## Stem cell control and dynamics

Stem cells are defined by their ability to self-renew and to give rise to differentiated cells. In the development of multicellular organisms, stem cells produce a multitude of cells and cell types. After fertilization, a blastocyst is formed that subsequently develops into an embryo. The blastocyst inner cell mass contains pluripotent cells, which are capable of forming the complete body. These stem cells differentiate into tissue-specific progenitors that generate all cell types within a tissue. Some of these stem and progenitor cells are transiently present during development, whereas others remain throughout the lifetime of an organism. Formation of tissues involves complex coordination of proliferation and differentiation of stem cells and tissue progenitors. Cells from the inner cell mass can be cultured *in vitro* as embryonic stem cells (ESCs) (Martin, 1981). These ESCs recapitulate self-renewal and differentiation *in vitro*, and are used to study stem cells and tissue formation (Cao et al., 2011; Gouti et al., 2014; Meinhardt et al., 2014; van den Brink et al., 2020).

After development, tissue homeostasis requires tissue-specific adult stem cells that maintain the cell population. Similar to developing tissues, stem cells in homeostasis require complex coordination of proliferation and differentiation to balance out natural cell death. It is widely recognized that a sub-population of developmental tissue progenitors become gradually restricted to a specific lineage and continue to maintain the tissue throughout life as adult stem cells (Nigmatullina et al., 2017). The transition from growth to maintenance is coordinated by a shift in the equilibrium of proliferation and differentiation within these developmental tissue progenitors (Berg et al., 2019; Guiu et al., 2019). In addition, in adults, the balance of proliferation and differentiation within the stem cell pool can be adjusted if needed. Upon injury, for example, proliferation has to increase to allow tissue renewal to fully restore its function. This is illustrated by axolotl limb regeneration, when amplification of the stem cell pool leads to formation of a blastema, a group of cells able to proliferate and replenish all cell types of the missing tissue (reviewed by Vieira and McCusker, 2019). Generation and amplification of the stem cell pool is achieved by dedifferentiation of cells within the injured tissue (Gerber et al., 2018).

When comparing development and homeostasis, differences can be observed in cell cycle rates of different developmental progenitors and adult tissue stem cells. One defining feature of adult tissue stem cells has historically been the existence of quiescence, which is a reversible state of cell cycle exit (Orford and Scadden, 2008). Quiescence was therefore a major difference between stem cells in development and homeostasis. However, nowadays quiescence is considered not as an independent characteristic but merely as an adaptation in stem cell regulation (Lahmann et al., 2019; Sueda et al., 2019). Even though division rates differ, developmental tissue progenitors and adult tissue stem cells are mainly restricted to the same lineage.

Although there are disparities between neural progenitors during development and homeostasis in lineage restriction, transcriptome, division rates and niche morphology (Morales and Mira, 2019), evidence for the differences between other developmental progenitors and adult tissue stem cells remains scarce. Thus, the fundamental principles of progenitors and adult stem cells, i.e. multipotency and longevity, are essentially identical, with differences in the balance between proliferation and differentiation. To examine the similarities between progenitors in development and stem cells in adult tissues, transplantations (including those between developing embryos and adult tissues, and vice versa), lineage tracing (Guiu et al., 2019) and single-cell sequencing should be performed. Such techniques provide information on the similarities in stem cell potency and specific transcriptome identity between development and homeostasis. The similarities between progenitor and adult stem cells can also inform the identification of tissue-specific progenitor and stem cells in embryonic and adult tissue. Although many types of quiescent adult stem cells have been detected as long-term DNA label-retaining cells (Cotsarelis et al., 1990; Wilson et al., 2008), this method cannot detect progenitors in the developing embryo due to the long incubation time that is required to observe label retention. Alternatively, stem and progenitor cells are found by lineage tracing and transplantation (Barker et al., 2007; Cambray and Wilson, 2002). The identification of specific markers that label tissue progenitor cells in embryos has traditionally been more difficult than in adult tissues, owing to the lower amount and (occasional) transience of progenitor cells present within embryos. Based on the assumption that embryonic progenitor cells persist into adulthood (Nigmatullina et al., 2017), one approach to find embryonic progenitors can be to test adult stem cell markers as potential markers in embryos. New computational approaches in the analysis of single-cell RNA sequencing (scRNAseq) data have allowed the identification of known and the prediction of new adult stem cell pools from tissue-specific scRNAseq datasets (Grün et al., 2015, 2016). Such approaches should be applied to embryonic tissue to predict the identity of progenitor cells.

## Cell fate decisions

Regulation of cell divisions and fate decisions are important, and therefore tightly regulated during both development and homeostasis. Upon stem cell division, the two daughter cells either perpetuate the stem cell state or differentiate, or one daughter cell remains a stem cell while the other daughter cell differentiates.

**Fig. 1.**
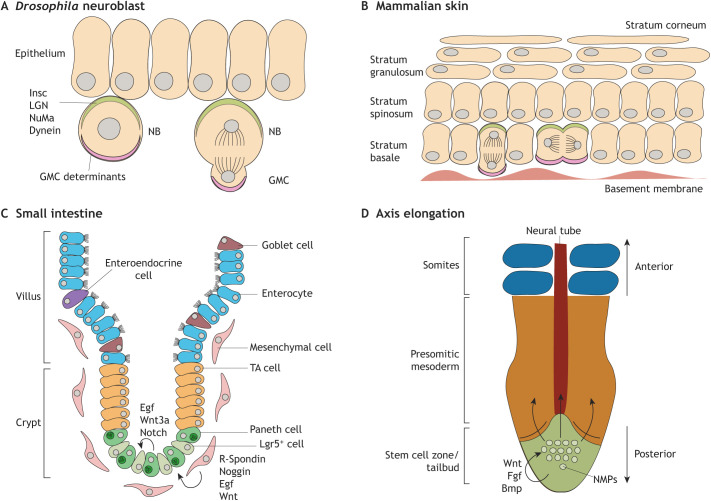
**Strategies for cell fate determination.** (A) A *Drosophila* neuroblast determines daughter cell fate by asymmetric localization of cell fate determinants. Initial polarization of Inscuteable (Insc), LGN, Nuclear mitotic apparatus protein (NuMA) and Dynein leads to eventual asymmetric localization and, hence, to asymmetric cell fate. GMC, ganglion mother cell; NB, neuroblast. (B) In the mammalian skin, polarity of stem cells is required for proper cell fate determination. However, no specific cell fate determinants have been found. Initial polarity is created by homologs of Insc, LGN, NuMA and Dynein. (C) Lgr5^+^ adult stem cells that maintain the small intestine during homeostasis reside in a niche located in the bottom of the crypt. Both Paneth cells and mesenchymal cells provide niche factors for stem cell maintenance. R-Spondin, Noggin, Egf and Wnt are secreted by mesenchymal cells, whereas Egf, Wnt3a and Notch are provided by the Paneth cells. (D) Neuromesodermal progenitors (NMPs), which give rise to the neural tube and the presomitic mesoderm, reside in a niche at the posterior tip of the vertebrate embryo. The source of niche factors Wnt, Fgf and Bmp remains to be elucidated.

Two different strategies to organize daughter cell fates after stem cell divisions exist. Cell fates can be defined intrinsically prior to division by asymmetric distribution of cell fate determinants during cytokinesis. Alternatively, position-dependent external factors can influence daughter cell differentiation. A thoroughly studied model to examine asymmetric distribution of cell fate determinants is the *Drosophila* neuroblast, the developmental progenitor of the central nervous system (Fig. 1A) (Gallaud et al., 2017). A characteristic of the *Drosophila* neuroblast is the ability to polarize proteins, including Inscuteable (Insc), LGN, Nuclear mitotic apparatus protein (NuMA) and Dynein, on the cell membrane prior to mitosis. Initial cell polarization is established by maintaining cell-cell contact to the neuroepithelium on one side. Polarization is followed by mitotic spindle alignment along the polarity axis, leading to asymmetric segregation of cell fate determinants. When the axis of division is perpendicular to the polarity axis, both daughter cells perpetuate the stem cell state. Similar to *Drosophila* neuroblasts, mouse epidermal progenitors maintain contact with the basement membrane to establish initial polarity during development (Fig. 1B) (Poulson and Lechler, 2010; Williams et al., 2011). Polarization of mouse homologs of Insc, LGN, NuMA and Dynein is required to direct the division axis. Loss of polarity in the mouse epidermal progenitors leads to issues in skin stratification and improper differentiation (Lechler and Fuchs, 2005). Polarity is essential; however, there is no direct evidence that asymmetric inheritance of intrinsic determinants drives cell fate decision upon division in the developing mammalian skin (Williams et al., 2011). In addition, the importance of external signalling in cell fate decisions cannot be excluded. In adult life, mammalian skin sheds and needs to be replaced continuously. Owing to the similarities between development and homeostasis of the skin, the regulation of stem cell differentiation is likely to be similar. With the development of epidermal skin organoids (Boonekamp et al., 2019; Lee et al., 2020), it is now possible to perform more-detailed analyses of the molecular mechanisms of skin stem cell divisions. Epidermal skin organoids are easily perturbable and are a more simplified research model than adult mouse skin. Deriving organoids from stem cells can be used to study development as well as homeostasis of this *in vitro* system. Current advances in organoid technology allow high-throughput approaches, such as protein labelling, perturbation screens and detailed expression analysis using RNA-sequencing (Artegiani et al., 2020; Gehart and Clevers, 2019; Phan et al., 2019). Furthermore, high-resolution live imaging allows addressing the function of intrinsic factors in regulating asymmetric divisions of stem cells. Finally, the importance of extrinsic factors could also be examined by perturbing the microenvironment.

Unlike asymmetric distribution of cell fate determinants during division, daughter cell fates can also be established by external signalling. External signalling implies that stem cell divisions are symmetrical and that daughter cell differentiation is based on the surrounding microenvironmental cues. Signalling inside the stem cell compartment maintains the daughter cell in a stem cell state: when the stem cell moves away from the stem cell compartment and from its microenvironmental cues, it differentiates. For a wide variety of tissues, adult stem cells are maintained by microenvironmental factors in a niche. For example, hair follicle stem cells, intestinal stem cells and hematopoietic stem cells keep their stem cell fate in a specifically developed niche (Aoki et al., 2016; Ge et al., 2020; Matos et al., 2020; Ouspenskaia et al., 2016; Sato et al., 2011; Schofield, 1978; Yang et al., 2017). The small intestine can be subdivided into two compartments: the villus, which contains differentiated cells; and the crypt, where stem cells reside (Fig. 1C). In homeostasis, the turnover time of the villi is 5 days. During homeostasis of the small intestinal epithelium, a highly proliferative stem cell pool is present that needs to differentiate continuously (Barker et al., 2007). Leucine-rich repeat-containing G-protein-coupled receptor 5 (Lgr5) marks these adult stem cells, which are surrounded by Paneth cells and mesenchymal cells in the crypt. The latter cell type provides niche factors for stem cell maintenance. Wnt, epidermal growth factor (Egf) and Notch signals are provided by the Paneth cells, whereas Wnt, Egf, R-Spondin and a bone morphogenetic protein (Bmp) antagonist, are provided by the mesenchymal cells (reviewed by Gehart and Clevers, 2019). The stem cell niche is restricted in size and can only contain a certain number of Lgr5^+^ stem cells. Therefore, competition between stem cells arises in which stem cells that are pushed out of the niche differentiate into cell types of the intestinal epithelium (Corominas-Murtra et al., 2020; Snippert et al., 2010). Here, homeostasis is achieved by external regulation on the stem cell population via external factors instead of regulating division symmetry in individual stem cells.

External regulation on the stem cell population similarly occurs in development. During embryonic development, somites, segments that later become muscles and vertebrae, and neural tube are formed after gastrulation. At this stage, the posterior tissue can be broadly subdivided into two compartments: first, the tailbud that contains a transient pool of progenitor cells; and second, the neural tube flanked by presomitic mesoderm (PSM) that contains differentiating cells (Fig. 1D). The neural tube and PSM are thought to originate from a bipotent progenitor: the neuromesodermal progenitor (NMP) (Tzouanacou et al., 2009). The co-expression of the mesodermal marker Brachyury and the universal neural progenitor marker Sox2 distinguishes NMPs. During vertebrate somitogenesis, NMPs are exposed to niche factors that are expressed in the tailbud (Wymeersch et al., 2019). During axial elongation, NMPs differentiate by exiting the tailbud into PSM and neural tube. In this way, a single NMP contributes to the anterior-posterior (AP) axis of the elongating tissue (Brown and Storey, 2000). When all NMPs have differentiated, this signifies the end of axial elongation (Wymeersch et al., 2016). NMP cell division is thought to be symmetrical, but daughter cell fates can differ based on the presence of niche factors. NMPs that move out of the stem cell niche prior to division will differentiate and no longer contribute to further tailbud lineages (Attardi et al., 2018; Tzouanacou et al., 2009). Overall, in diverse tissues, both during development and tissue homeostasis, stem cell control can be guided by external niche factors that control stem cell behaviour on a population level. The specific signalling required to maintain the NMP fate includes Wnt, fibroblast growth factor (Fgf) and Bmp (reviewed by Henrique et al., 2015). Although these signalling requirements are known for NMPs, the exact origin of the signals is unknown. To find the cells that form the NMP niche, the approach used to reveal the small intestine stem cell niche could serve as a template. Here, a combination of microarray analysis, *in situ* hybridization and cell ablation was used to identify, locate and validate the stem cell niche (Sato et al., 2011; Valenta et al., 2016). Recent advances in single-cell sequencing and mRNA visualization techniques allow for more in depth and higher resolution analysis of the NMP niche (Choi et al., 2018; Hwang et al., 2018). Thus, in both embryonic development and adult tissue homeostasis, stem cell proliferation and fate decisions can be controlled either at the single-cell level, e.g. via asymmetric cell division, or at the population level by external signalling within a niche.

## Self-organization of tissues

Besides regulation of cell fates at single-cell level, higher order tissue arrangement is necessary to form a multicellular organism. To form sophisticated tissue patterns, spatiotemporal organization of cells during development is required (Box 1). Embryogenesis starts from a single zygote that gradually develops into a complete body. Self-organization of cells, defined by interacting cells that coordinate themselves into larger scale patterns, is key to establish proper tissues. Crucial processes of self-organization are initial symmetry-breaking events and collective rearrangement of cells. During self-organization, regulation of stem cell proliferation and differentiation directs the tissue outline, resulting in the proper arrangement of all organs.

### Symmetry breaking

**Fig. 2.**
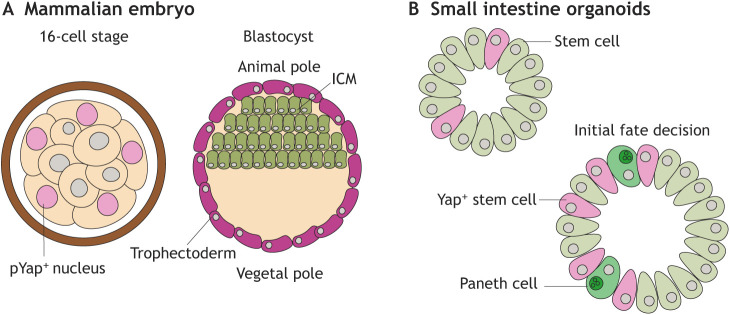
**Symmetry-breaking events.** (A) Establishment of an apical domain in outer cells induces symmetry breaking from the outside to the inside of the early mouse embryo. Nuclear phosphorylated Yap (pYap) then induces activation of Cdx2 and the first lineage segregation into trophectoderm and inner cell mass (ICM). (B) Small intestine organoids are initially spherically symmetrical and break symmetry after withdrawal of Wnt supplements from the medium. Fluctuation in Yap1 signalling organize the formation of Paneth cells, the first differentiation of the maturing organoid.

In the initial steps of self-organization, it is necessary to establish an asymmetric cell population and, therefore, an initial break in population symmetry is required. Symmetry breaking can be induced by random fluctuations in gene expression or by external factors that induce a heterogenous population. In mammalian embryos, symmetry is broken during the first lineage segregation, during which trophectoderm and inner cell mass (ICM) are established. Research has implicated the importance of Yap1 signalling, which is active in the outer cells of the embryo, in initiating the Cdx2 transcription necessary for trophectoderm formation (Fig. 2A) (Anani et al., 2014; Maître et al., 2016; Strumpf et al., 2005). The Hippo pathway, which includes Yap1, is important in cell adhesion and polarity. Both cell adhesion and polarity are crucial for symmetry breaking; however, the exact origin of Yap1 during the initial symmetry break remains elusive (Korotkevich et al., 2017). Following ICM specification, a further subdivision occurs into epiblast and primitive endoderm. Extra-embryonic tissue induces primitive streak formation in the epiblast: the first symmetry-breaking event of the anterior-posterior (AP) axis (Rodriguez et al., 2005). Gastrulation occurs at the primitive streak, where cells from the epiblast internalize and form the mesodermal germ layer. Symmetry breaking remains a poorly understood and difficult to study process *in vivo* that requires further analyses. However, several *in vitro* models have been established that can be used to study the symmetry-breaking processes, such as embryoid bodies (Sagy et al., 2019), blastoids (Rivron et al., 2018), gastruloids (Turner et al., 2017) and neuroephitilial cysts (Meinhardt et al., 2014). All these models are initially spherically symmetric. Therefore, symmetry breaking and subsequent self-organization have to occur to drive development of these systems. In systems without predetermined polarity, stochastic fluctuations in gene expression can cause an initial symmetry break.

During homeostasis, symmetry breaking is usually not required because the main function of stem cells is the maintenance of an existing structure. However, adult stem cells retain their symmetry-breaking capacity, as recapitulated by intestinal organoid formation (Sato and Clevers, 2013). Plating out single stem cells results in spherically symmetric organoids. After 72 h, withdrawal of Wnt supplements from the medium allows the differentiation of stem cells. The first differentiation step is a symmetry-breaking event that allows Paneth cells to form, which drives the organization of crypt structures within the organoid (Serra et al., 2019). Although growth of organoids from single cells is rather considered to be comparable to tissue regeneration (where symmetry breaking is necessary), it highlights the similar potential for embryonic and adult stem cells to break symmetry. It has been shown that transient activation of Yap1 is necessary for symmetry breaking, similar to the symmetry-breaking event in the first lineage segregation of trophectoderm and ICM (Fig. 2B) (Hirate et al., 2013; Nishioka et al., 2009; Serra et al., 2019).

The mechanism of how symmetry breaking occurs, and the issue of whether there is a general mechanism of symmetry breaking in development and regeneration in different species, is not understood. Yap1-based signalling seems to be a generally occurring early event in symmetry breaking. The involvement of mechanics, surface tension and location within a three-dimensional structure in this process should be investigated in the different model systems, such as the regenerating axolotl or hydra, akin to studies in, for example, early mouse embryos (Maître et al., 2016; Samarage et al., 2015). With present *in vitro* models of mammalian development and homeostasis, symmetry breaking can be investigated at subcellular and molecular levels. For example, with the more easily perturbable organoid, gastruloid and blastoid systems (Rivron et al., 2018; Sato et al., 2011; van den Brink et al., 2014), the specific genes involved in symmetry breaking can be unravelled.

### Collective cell rearrangements

After symmetry breaking, signalling centres are established that continue to guide self-organization processes such as patterning and collective cell rearrangements. Collective cell migration is a major component of tissue organization, as progenitors are often specified at a distance from their final position. An example of collective behaviour during development is the migration of primordial germ cells (PGCs) in mice. After gastrulation, PGCs migrate from the posterior primitive streak to the endoderm. PGC pool expansion occurs during anterior migration over the hindgut. When PGCs reach the height of the future gonads, migration continues bilaterally over the dorsal mesenterium and genital ridges. Chemokines function as attractants and repellents to organize this sophisticated form of cellular organization (Nikolic et al., 2016). Both, Bmp and transforming growth factor β (Tgfβ) have been implicated in directional PGC migration to the genital ridges (Dudley et al., 2007, 2010).

In homeostasis, collective cell behaviour in the form of active migration is rather used to arrange stem cells and differentiated cells within adult tissues. In the small intestines, epithelial cell migration up the villus has classically been thought to be directed by a passive pushing force generated by mitoses in the crypt, which generates pressure towards the villi. Recently, it has been shown that crypt-generated pushing forces only affect epithelial cells as far as the base of the villus and that further migration to the top of the villus is an active form of migration (Krndija et al., 2019). The attractant in this scenario is still unknown; nonetheless, Bmp or Tgfβ (both of which have been implicated in PGC migration) could be involved. Tgfβ and Bmp ligands are present in a top-to-villus base gradient, which could attract the active migration towards the top of the villus (Haramis et al., 2004; Winesett et al., 1996).

Symmetry breaking and collective behaviour of cells within a tissue are key for establishing and maintaining tissue organization. Migration of, for example, immune cells has been studied *in vitro* by applying external gradients of chemokines. This allowed the in-depth analysis of required signals and intracellular processes (Vesperini et al., 2021). Collective cell migration and cell rearrangement of, for example, PGCs and cells in the small intestine could be studied in similar systems using microfluidic chips (Gupta et al., 2021; Nikolaev et al., 2020). Recapitulating these processes *in vitro* will allow the dissection at mechanistic and subcellular levels. Although detailed steps of these processes remain to be elucidated, the fundamental concept of tissue organization in development and homeostasis appears to be similar.

## Pattern formation and maintenance

**Fig. 3.**
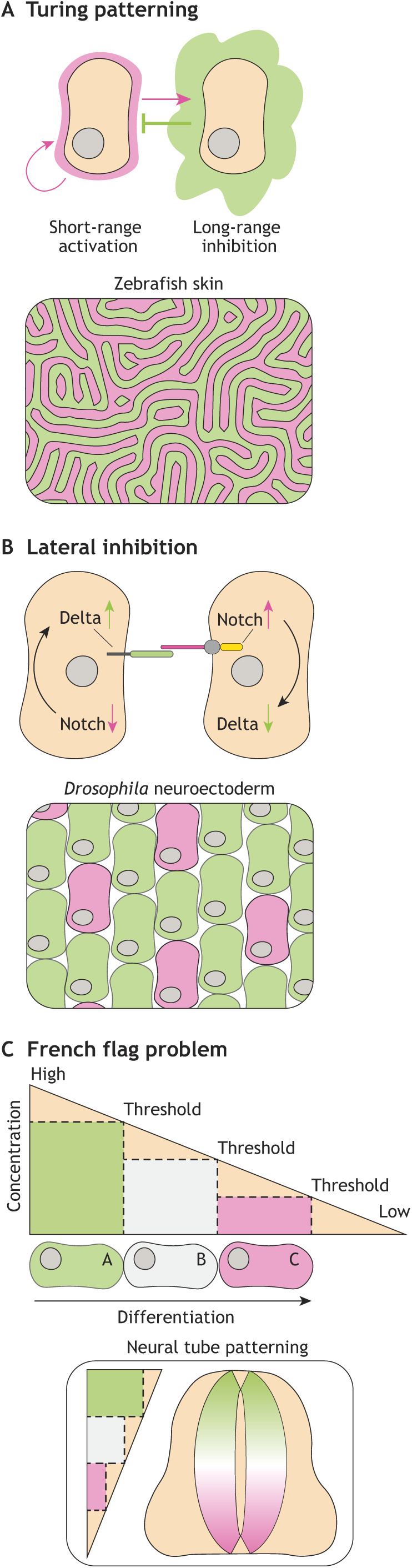
**Models for patterning.** (A) Turing patterning is based on secretion of short-range activators and long-range inhibitors. By using of this mode of patterning, complex organization of cell types can occur, such as the labyrinth patterning of zebrafish skin. (B) Lateral inhibition is created by Notch signalling. Notch signalling is activated by the membrane-bound ligand Delta. Active Notch signalling results in downregulation of Delta and therefore inactive Notch signalling on neighbouring cells. Conversely, low Notch signalling results in high Delta and active Notch signalling in neighbouring cells. For example, lateral inhibition creates a salt-and-pepper pattern of cell fates in the *Drosophila* neuroectoderm. (C) The French flag problem is a gradient-based model of patterning. Information is encoded in the strength of the gradient. Cells positioned at different locations within a gradient receive different signals and therefore behave/differentiate differently. For example, a neuronal pattern arises based on the position of neuronal precursors within a gradient.

Tissue patterns, such as spots or stripes on the skin, are established during development and maintained throughout adult life. Within a patterned tissue, cells receive signals and acquire identities based on their position (Box 1). Different approaches exist to manifest tissue patterns (Fig. 3). First, the Turing patterning system (Turing, 1952) depends on source cells secreting activators and inhibitors with different diffusion co-efficients. By secreting local activators and long-range inhibitors, tissues arrange into a Turing pattern (Fig. 3A). The skin of zebrafish is characterized by alternating gold and silver stripes. During development, melanophore and xanthophore pigment cells organize in a Turing-type pattern by having two opposing effects on each other dependent on the distance (Nakamasu et al., 2009). Inhibition occurs locally, whereas activation occurs over a long range. Another approach to create patterns is via lateral inhibition (Fig. 3B). A well-studied example is Notch lateral inhibition between cells of the *Drosophila* neuroectoderm (Kunisch et al., 1994). This patterning is based on the interaction of the Notch receptor and the membrane-bound ligand Delta on neighbouring cells. Active Notch signalling represses the ability of a cell to express Delta, which therefore cannot activate Notch in neighbouring cells. A stable salt-and-pepper pattern of cell identity arises in the neuroectoderm, where cells high in Notch signalling are surrounded by cells low in Notch signalling. The third patterning model is described by the ‘French flag problem’ (Fig. 3C) (Wolpert, 1969). A model proposed by this problem is based on morphogen gradients that pattern the tissue. Cells within different parts of the gradient receive different amounts of signal. Information encoded in the strength of the signal, defines the cellular outcome, as exemplified by neuronal patterning within the neural tube (Briscoe and Small, 2015). The characteristics of gradients are influenced by morphogen synthesis, diffusion and degradation (Inomata, 2017; Wolpert, 2002).

### Gradient-based pattering

**Fig. 4.**
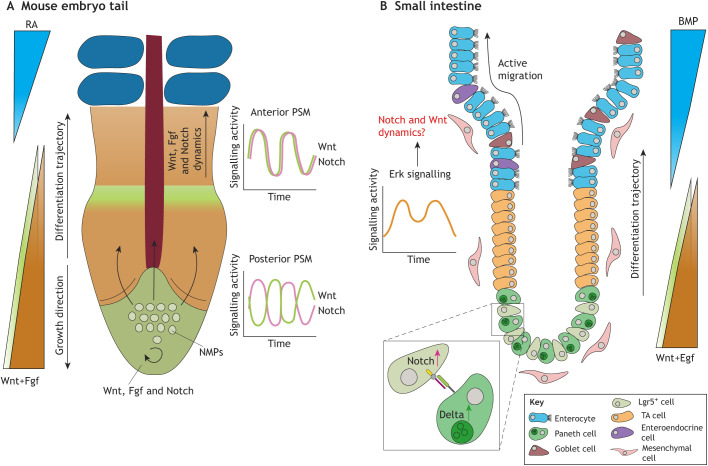
**Gradients and signalling dynamics during somitogenesis and small intestine homeostasis.** (A) Somitogenesis is coordinated by posterior gradients of Wnt and Fgf, and an anterior antagonistic gradient of retinoic acid (RA). Neuromesodermal progenitors (NMPs) from the posterior migrate along the posterior gradient, and concomitantly differentiate and form somites when they reach the determination front. Further patterning is achieved by signalling dynamics. Somites form in a rhythmic manner and periodicity is determined by oscillatory activation of Wnt, Fgf and Notch signalling. In the posterior presomitic mesoderm, Wnt and Notch signalling oscillate out of phase, while in the anterior, Notch and Wnt signalling are synchronized. (B) The small intestine is patterned by many gradients, including Wnt and Egf gradients originating from the crypt side and an antagonistic Bmp gradient originating from the villus. Lgr5^+^ adult stem cells progress into transit amplifying (TA) cells and finally differentiate into their terminal state. This differentiation trajectory occurs along the crypt side gradient. Lgr5^+^ stem cells are maintained in the crypt by lateral inhibition of Notch by Paneth cells. Although gradients and lateral inhibition are found to be necessary during small intestinal homeostasis, the importance of signalling dynamics remains elusive. Pulsatile Erk dynamics have been found during organoid formation (Muta et al., 2018); however, dynamics within the Notch and Wnt signalling pathways are yet to be explored.

Gradient-based patterning can occur only after an initial symmetry-breaking event and establishment of signalling centres. During development of all tissues, gradients are necessary for proper specification of cell identities. Gradients subdivide tissues into domains of differentiation, which allows reproducible tissue architectures within organs, and between organisms and species. In the mouse PSM, gradients of Wnt and Fgf pathway activation originate from the posterior domain, whereas a counteracting gradient of retinoic acid (RA) originates from the somites (Fig. 4A) (Aulehla and Pourquié, 2010). Progenitor cells that reside in the tailbud receive high Wnt and Fgf signalling, while cells outside the tailbud follow a differentiation trajectory along these gradients. Cells moving anteriorly continuously receive less Wnt and Fgf, and mature until they reach the most anterior domain of the PSM. There, cells are exposed to high RA and low Wnt/Fgf, which induces them to differentiate into somites (Dubrulle et al., 2001).

Co-expression of Fgf/Egf and Wnt gradients is important in many tissues both during development and homeostasis (Harshuk-Shabso et al., 2020; Tang et al., 2019; ten Berge et al., 2008). A well-studied murine homeostatic model, in which co-expressed Wnt/Egf gradients are present, is the small intestine (Farin et al., 2016; Yang et al., 2017). In small intestine homeostasis, Wnt/Egf signalling is high in the bottom of the crypt and an antagonistic (Bmp) gradient originates from the top of the villi (Fig. 4B). The crypt domain preserves the stem cells, whereas the villus domain defines epithelial differentiation (Qi et al., 2017). Cells that migrate along the Wnt/Egf gradient undergo a differentiation trajectory, meaning maturation occurs when the gradient diminishes. Transit-amplifying (TA) cells divide and migrate until the antagonistic Bmp gradient is reached, which allows for epithelial differentiation (Beumer et al., 2018).

In homeostasis, patterns primarily need to be maintained but, after injury, the tissue pattern needs to be re-established during regeneration. TA and secretory cells in the small intestine can dedifferentiate to increase the Lgr5^+^ stem cell pool upon injury (Hsu et al., 2014; Jones et al., 2019; Murata et al., 2020; Yan et al., 2017). Subsequently, symmetric Lgr5^+^ stem cell divisions continue until crypt size restriction is reached and cells start to compete for crypt space again (Snippert et al., 2010). After stem cell recovery, differentiation along the redefined gradients replenishes all cell types and the tissue pattern is re-established.

Development and homeostasis both rely on signalling gradients for proper tissue formation and maintenance, respectively. How signalling gradients are generated and controlled, and how these are interpreted to control cellular identity is not understood in detail. Tools developed to examine gradient functionality should therefore be applied to both fields. Specific modulations of, for example, the amplitude or range of a gradient are necessary to study the information encoded within a signalling gradient. Altering the gradient shape provides the opportunity to analyse the function of gradients for development or tissue homeostasis in more detail. Classically, heparin beads have been soaked in ligand protein and grafted in close proximity to tissues to produce novel signalling gradients (reviewed by Aulehla and Pourquié, 2010). Another method to produce reoriented gradients is to genetically engineer a ligand gene to be expressed by a different promoter. Hereby, the source cells forming the gradient are changed (Gao et al., 2018). Whereas bead engraftment and genetic engineering distort tissue integrity or require time-consuming genome modifications, microfluidic devices are capable of recreating the tightly regulated microenvironment of signalling gradients found in tissues *in vitro* (Demers et al., 2016). Therefore, maintaining tissue integrity by using a microfluidic system and precise gradient adaptations can be achieved (Manfrin et al., 2019). Gradient shape can also be altered by modulation of ligand capture proteins. By specifically targeting the ligand capture proteins, the clearance of the ligand within the tissue can be increased or decreased, adjusting the diffusion coefficient and thereby the steepness of the gradient (Stapornwongkul et al., 2020).

### Signalling dynamics

During development and homeostasis, cell behaviour is instructed by a limited number of conserved pathways (Sonnen and Aulehla, 2014). To be able to convey accurate information to cells, information is not only encoded in gradients but also in the dynamics of signalling activation and gene regulatory networks. Early on, signalling dynamics have been proposed to provide additional layers of encoding information to compensate for the limited number of pathways present (Strogatz et al., 1994). Examples of specific features within signalling dynamics are duration, delay and strength of signal. Additionally, the phase, frequency and amplitude in oscillatory signalling can potentially cause different cellular outcomes. Recently, experiments have indicated that signalling dynamics exist and are important during stem cell differentiation in development (Manning et al., 2019; Seymour et al., 2020; Sueda et al., 2019). Technological improvements have created the ability to visualize and perturb intracellular signalling (e.g. Isomura et al., 2017; Sonnen et al., 2018; Yoshioka-Kobayashi et al., 2020). This now enables experimental approaches to examine signalling dynamics at the functional level. For example, murine somitogenesis is controlled by oscillations of Notch and Wnt signalling (Fig. 4A). In more detail, crucial information for periodic segmentation of the PSM is encoded in the phase correlation of Wnt and Notch signalling oscillations (Sonnen et al., 2018). Other pathway dynamics are observed in the PSM, including oscillatory activation of the Fgf/Erk pathway (Dequéant et al., 2006). The signalling dynamics of the segmentation clock are conserved between vertebrates, including humans (Diaz-Cuadros et al., 2019 preprint; Oates et al., 2012; Matsuda et al., 2020). The importance of signalling dynamics has been further studied *in vitro*: different Erk phosphorylation dynamics have been observed upon induction of differentiation into distinct lineages in mouse ESCs (Deathridge et al., 2019). In mouse pre-implantation embryos, lineage-specific Erk signalling dynamics underlie cell fate decisions (Pokrass et al., 2020; Simon et al., 2020).

However, signalling dynamics during adult tissue homeostasis remain sparsely explored. So far, differences in Erk signalling dynamics have been found in distinct differentiated epidermal cells using live imaging of murine skin (Hiratsuka et al., 2020). Furthermore, pulsatile Erk dynamics have been found in the small intestine; however, the function of these Erk pulses remains elusive (Muta et al., 2018). Homeostasis of the small intestine is maintained by similar signalling pathways that are present in somitogenesis. This raises the issues of whether and how signalling dynamics affect the cell turnover and differentiation during homeostasis of the small intestine and other homeostatic processes (Fig. 4B).

Specific tools can be applied to investigate signalling dynamics. To visualize dynamics, classic protein tags might not be ideal owing to fluorophore stability and maturation time. Specific tags have been developed that are destabilized, fast maturing or cause re-localization; these provide the opportunity to visualize the dynamics of signalling at a high temporal resolution (Bothma et al., 2018; Diaz-Cuadros et al., 2019 preprint; Yoshioka-Kobayashi et al., 2020). To visualize the dynamics of protein phosphorylation, fluorescence resonance energy transfer (FRET) biosensors have been used (Deathridge et al., 2019; Kuijt et al., 2020; Ponsioen et al., 2021). Changes in phosphorylation status of the biosensor confer a direct fluorescent readout for the functionality of the protein of interest. A method to look at dynamic protein interactions – besides FRET-based sensors – is the use of split proteins. In this process, two proteins of interest are tagged and fluorescence occurs only when the proteins are in close proximity to each other (Tebo and Gautier, 2019). To functionally assess the role of signalling dynamics, modulation of signalling dynamics is necessary. Microfluidic and optogenetic systems allow for spatiotemporal perturbation in *ex vivo* and *in vitro* culture systems (e.g. Isomura et al., 2017; Kellogg et al., 2014; Manfrin et al., 2019; Sonnen et al., 2018; Toettcher et al., 2011). With such recent technological developments for visualization and perturbation, the function of signalling dynamics in stem cells can now be studied in detail during both development and homeostasis.

## Perspectives

Here, we have discussed various examples that highlight the similarities between developing and adult tissues. These examples imply that general principles underlie both development and homeostasis with the differences lying in essential details, e.g. the balance between proliferation and differentiation.

To address remaining unanswered questions in either field, it is very sensible to compare and investigate the process in question at several stages and in several types of multicellular systems. This includes the study of available literature, the application of established tools and technologies, and the direct experimental comparison of a process in either field. Tools such as genetically engineered animal models, *in vitro* stem cell-based model systems, genetic constructs, microfluidic systems, cell tracking, single-cell sequencing and bioinformatic analyses can be widely used in various research fields. As an example of a direct experimental comparison, when a new *in vitro* organoid model is being developed, it makes sense to explore what is known about factors regulating its *in vivo* development. This knowledge can be used as a starting point to define a growth factor cocktail for organoid maintenance. Another unanswered question is ‘which factors are involved in stem cell self-renewal during development?’. *In vitro* models are applied for the identification of signals to maintain stem cell potency. Although stem cell niches are continuously present in homeostasis, making it easier to study factors required for self-renewal (Sato et al., 2011), some developmental progenitors and their niche are present only transiently (Wymeersch et al., 2019). *In vitro* models that require stem cell maintenance, such as organoids, have been crucial for unravelling factors required for stem cell maintenance or differentiation during homeostasis. Therefore, *in vitro* models for developing tissues, such as gastruloids, can aid in the identification of niche factors required for stem cell maintenance and differentiation during development. In addition, to resolve the underlying mechanisms of symmetry breaking, *in vitro* models of both development (van den Brink et al., 2014) and homeostasis (Serra et al., 2019) will be useful. Applying very similar tools has, for example, identified Yap-Taz signalling as a crucial factor controlling symmetry breaking in both early embryos and small intestines (Maître et al., 2016; Serra et al., 2019).

Other outstanding questions include ‘how are morphogen gradients established and maintained?’ and ‘how is the size and scaling of tissues controlled?’, which is fully understood neither in developing embryos nor in homeostatic tissues. Tools for external modulation of signalling gradients, such as microfluidic set-ups (Sonnen and Merten, 2019), can be applied to address these questions in both developing and adult tissue. However, the results will be relevant for both research fields. In the context of patterning, an upcoming research field is the importance of signalling dynamics. Recent advances in visualization and perturbation of signalling dynamics allows for functional studies during both development and homeostasis.

The general principles guiding development can also be applied to tissue regeneration due to the resemblance of tissue regeneration to development (Gerber et al., 2018; Honkoop et al., 2019). Therefore, regeneration can be viewed as a temporary reversion to the developmental state. The overlap between regeneration and development creates the opportunity to address both fields similarly. For example, new technical improvements allow examination of signalling dynamics and their importance in regeneration.

In cancer, many of the previously described principles are deregulated. Cancer is defined by cells proliferating at uncontrolled speed, cells losing responsiveness to external signals and loss of proper tissue pattern (Hanahan and Weinberg, 2011). In the initial steps of carcinogenesis, cancer cell mutations cause a shift in the equilibrium of proliferation and differentiation towards a high proliferative state with perturbed differentiation. Cancer cells have lost dependency on a niche due to mutations hijacking self-renewal pathways (Drost et al., 2015; Fumagalli et al., 2020). Alternatively, cancer cells maintain their own microenvironment (Lim et al., 2017; Tammela et al., 2017). Cells that do differentiate within a tumour lose their proliferative capacity and are no longer tumorigenic (Cheng et al., 2020). In addition, signalling dynamics have been shown to specify tumour traits in the intestine (Muta et al., 2018; Ponsioen et al., 2021). The possibility that dysregulation of signalling dynamics in cancer underlies tumorigenic properties makes it an interesting, yet to be investigated, research question. The requirement for activated self-renewal illustrates that, although most regulatory mechanisms are overwritten in cancer, general mechanisms of development and homeostasis, such as stem cell control, still apply.

General principles therefore exist that guide both embryonic development and tissue homeostasis. To leverage this fact, researchers should broaden their horizons and consciously promote interactions between the different disciplines. Meetings focussing on such general principles can help to bring together researchers studying stem cells and patterning in development and tissue homeostasis, for example. These principles can also be applied to tissue regeneration and cancer biology. Bridging research of stem cell control, self-organization and patterning between the different fields has direct clinical implications and opens new therapeutic opportunities to induce tissue regeneration or counteract cancer in the future.
